# Rotational Rheology of Wood Flour Composites Based on Recycled Polyethylene

**DOI:** 10.3390/polym13142226

**Published:** 2021-07-06

**Authors:** Antonella Patti, Gianluca Cicala, Stefano Acierno

**Affiliations:** 1 Department of Civil Engineering and Architecture (DICAr), University of Catania, Viale Andrea Doria 6, 95125 Catania, Italy; gianluca.cicala@unict.it; 2 Department of Engineering, University of Sannio, Piazza Roma 21, 82100 Benevento, Italy

**Keywords:** wood-polymer composites (WPC), recycled thermoplastics, torque measurements, rheological properties, dynamic mechanical analyses

## Abstract

In this paper, we study the effect of the addition of wood flour as a filler in a recycled polyethylene (r-PE) in view of its potential applications in 3D printing. The composites, prepared by melt mixing, are characterized with torque measurements performed during the compounding, dynamic rotational rheology, and infrared spectroscopy. Data show that the introduction of wood results in increased viscosity and in sensible viscous heating during the compounding. The r-PE appear to be stable at temperatures up to 180 °C while at higher temperatures the material shows a rheological response characterized by time-increasing viscoelastic moduli that suggests a thermal degradation governed by crosslinking reactions. The compounds (with wood loading up to 50% in wt.) also shows thermal stability at temperatures up to 180 °C. The viscoelastic behavior and the infrared spectra of the r-PE matrix suggests the presence of branches in the macromolecular structure due to the process. Although the addition of wood particles determines increased viscoelastic moduli, a solid-like viscoelastic response is not shown even for the highest wood concentrations. This behavior, due to a poor compatibility and weak interfacial adhesion between the two phases, is however promising in view of common processing technologies as extrusion or injection molding.

## 1. Introduction

Plastics are one of the main components of products of everyday life and industrial activities, such as packaging, agriculture, automotive, and biomedical applications, becoming an essential element for the way of life. As a consequence, an increase in plastic production and plastic products has been verified in recent times. Conventional plastics are materials with (relatively) high strength and durability and requiring hundreds of years to break down under normal ambient conditions. This represents an important disadvantage from the perspective of environmental impact and aspects of pollution [1]. 

As reported by Singh and Sharma [2], over 300 million metric tons of plastic are produced each year and half of that amount is spent on disposal applications, i.e., activities lasting less than one year: the product is used and thrown away one year after purchase. The accumulation of solid plastic waste in the environment has become an increasingly important worldwide problem to consider and deal with [3].

Over the past decades, environmentalists have devoted more and more effort to the impact of chemical and industrial processes and, as a consequence, in a number of countries, governments have promoted rules and laws to protect the quality of the environment for the future [4]. In this context, chemical industries have been pushed to adopt non-polluting chemical processes and materials, reduce the use of hazard chemicals, efficiently use raw materials, and reduce emissions and wastes. This has translated into a growing interest in biodegradable renewable systems, such as composites reinforced with plant fibers [5].

Natural or lignocellulosic materials, such as jute, coir, rice husk, bagasse, or sisal have been taken into account for the production of “green” composites, by replacing the traditional synthetic (glass or carbon or aramid-based fibers), or common fillers (calcium carbonate, silica, or talc), in widespread used thermoplastics (polyethylene, polypropylene, and polystyrene) [6].

As cost-effective natural filler, wood has often adopted in the plastics industry to improve the strength or stiffness of thermoplastics, to reduce the costs, to enhance recyclability and eco-compatibility, or diminish the wear on processing equipment. In this perspective, wood-plastic composites were early used for exterior non-structural or semi- structural building products in the civil engineering (such as decking, fencing, siding, window frames, and roof tiles [7]) or also actualized for various industries, including automotive, household items, packaging, and consumer goods or living supplies as yard products [8].

A wood-plastic composite (WPC) refers consists of wood-based elements (such as lumber, veneer, fibers, or particles) embedded in a polymer matrix. Typically, extrusion, thermoforming, compressive or injection molding have been adopted, as processing techniques, in the compounds preparation. Unfortunately, wood start to degrade at approximately 200 °C and this represents a limiting condition to the choice of the polymeric matrix in WPCs. Typical polymers, such as polypropylene (PP), polyethylene (PE), polystyrene (PS), and poly vinyl chloride (PVC), have been adopted in wood-polymer compounds [9].

For example, in work by Wang et al. [10], polypropylene (PP) composites reinforced with 15, 30, and 45 wt.% wood powder (WP) were prepared by injection molding and characterized in terms of thermal, mechanical, and dynamic mechanical properties. Experimental results show that the presence of wood in polypropylene determined on the one hand an increment of strength and rigidity but, on the other hand, the toughness of the compounds did not improve. However, the authors affirmed the potentiality of WP- PP composites to replace fiberglass-reinforced composites in construction, sports facilities, and the automotive industry. 

The influence of five types of plastics, high density polyethylene (HDPE), low density polyethylene (LDPE), polypropylene (PP), polyvinylchloride (PVC), and polystyrene (PS), on the mechanical properties of wood-composites have been studied by Ratanawilai et al. [11]. According to their data, WPCs based on PS and PP achieved the highest performance in terms of strength and weathering compared to the other matrices.

Additionally, recycled plastics were used for the preparation of wood-based composites; for example the effects of recycling operations on the mechanical and rheological properties of wood-flour in a blend of low and high density polyethylenes has been investigated by Habibi et al. [12]. A pre-mixing of the two polymers was found to increase flexural and tensile properties of the developed compounds. An increment in complex viscosity and storage modulus was measured in prepared samples by melt-premixing compared to those obtained by simultaneous compounding.

Despite the interesting features of WDCs systems, some difficulties came from the shaping through the traditional methods of extrusion and injection molding. Rheological characterization has been proven to be a very useful technique for the understanding of the manufacturing processes and to optimize process conditions and formulations in order to achieve good quality of the final products [13].

In recent years, the interest on the additive manufacturing (AM), also called 3D printing or rapid prototyping, has markedly concerned several fields [14]. The most popular procedure among 3D printing techniques is the so-called fused deposition modeling (FDM), during which extruded filaments are printed one on top the others following a geometric pattern into a three-dimensional structure, according to layer-by-layer deposition. On the one hand, the versatility offered by 3D printers, linked to a great reduction in equipment costs, have promoted a wide spread of this technology. On the other hand, critical issues regarding the environmental impact, coming from energy and material consumption, and wastes production, have arisen. Possible actions in order to improve the sustainability of additive manufacturing consist in the development of sustainable (eventually from recycling operation of waste products) for 3D printing [15]. A wide range of performance in 3D printed materials can be achieved with the right combination of polymer, filler, and additives. The commonly used thermoplastics available for FDM are acrylonitrile-butadiene-styrene (ABS), polylactic acid (PLA), and polyamide (PA), but frequently also polyamide or nylon (PA), polycarbonate (PC), poly-methylmethylacrylate (PMMA), polyethylene (PE), and polypropylene (PP). Nowadays, most researches are focused on composites and biocomposites, i.e., at least one component derived from biological or natural sources. The use of natural materials such as wood helps to reduce the use of petroleum-based plastics and, thus, the environmental impact. Different amounts of wood flour (up to 50 wt.%) were used as reinforcing filler in polylactide acid (PLA) by testing physical, mechanical, and rheological properties of the 3D printed composites [16]. The effect of extrusion temperature on the physical properties of the printed wood/PLA samples such as moisture content, surface roughness, water absorption rate, and thickness swelling rate, were studied in the work of Yang [17]. The 3D printable composite filaments have been made from cardboard dust and high density polyethylene (HDPE) [18]. Waste products have been proposed for 3D printing applications: post-consumer textile waste and polyethylene terephthalate (PET) water bottles were melt compounded to form a monofilament feedstock for extrusion-based 3D printing platforms [19]. 

In this framework, the purpose of this work is to explore the characteristics of a polymer filament based on recycled polyethylene in view of its potential applications as raw material in extrusion and 3D printed parts. The inclusion of wood flour in the chosen matrix is proposed in order increase the eco-compatibility and the overall performance of final products. At regard, rotational rheological tests were conducted on samples to investigate aspects related to the thermal stability of obtained compounds, and effects of powder content on polymer viscoelasticity. Torque and temperature measurements were also reported to examine the processability of formulations. Finally, spectroscopic analysis was used to confirm the thermal degradation of the tested materials.

## 2. Materials and Methods

A commercial low-density recycled polyethylene (r-PE), obtained by regenerating films coming from the greenhouse cover, supplied in pellets by ILAP (Ragusa, Italy) was used as matrix. A wood flour, micrometric in size, supplied from wood processing factory, was used as filler.

Composites, containing recycled polyethylene (r-PE) and 25, 35, and 50 wt./wt.% of wood (WD) flour as filler, were prepared by combining the two components in a batch mixer (Brabender Plastograph EC-Brabender GmbH and Co. KG, Germany). The mixing equipment was connected to a drive unit (torque rheometer) that allowed the collection of torque data and the thermal control of the mixing chamber during the compound preparation. The temperature of the mixing chamber measured through four thermocouples located in different points: in the center of the rotating screws (bulk temperature), near the rear wall, front wall, and mixer bowl. The bulk temperature and torque as a function of mixing time are provided by the software; when the set-point temperature is achieved by all the thermocouples, a torque calibration is done, and the chamber is filled (to about the 80% of its volume) pouring polymer pellets and wood flour. The compounding phase was conducted at a screw rotational speed of 30 RPM, a temperature of 180 °C, and a mixing time of 15 min. Before the composite preparation, the wood flour was dried overnight under vacuum in an oven at a temperature of 50 °C. The material that exited the mixer was reduced to fragments and extruded at 180 °C in a twin screw extruder (mod. KETSE 20/40 D, Brabender) to form a filament.

Viscoelastic characterization of molten composites was conducted using a strain-controlled rotational rheometer (mod. ARES-G2, by TA Instruments, New Castle, DE, USA), equipped with parallel plates (25 mm in diameter) geometry and a forced-convection oven for the temperature control. Preliminary strain sweep tests were performed in order to identify the linear viscoelastic region of the material. The materials were subjected to small–amplitude oscillations at a frequency of 1 rad/s and a strain amplitude of 1% for a duration of 1500 s (also called “Time sweep tests”) at four different temperatures, ranging from 160 °C to 220 °C, in order to verify the thermal stability of matrix and of the compounds. The linear viscoelastic characterization of the samples in the frequency range from 0.1 to 100 rad/s (the test are also called “Frequency sweep tests”) was performed at 160 °C, 170 °C, 180 °C. All the experiments were carried out on specimens under air atmosphere in order to assess the material behavior under real working conditions.

An infrared spectrometer (mod. Spectrum 65 FT IR, produced by Perkin Elmer, Waltham, MA, USA), was used in attenuated total reflection (ATR) modality to acquire the spectra of r-PE and r-PE-WD compounds. The samples were analyzed before and after a heat treatment conducted in an oven at 180 °C for 1 h. Afterward, the same samples were subjected to further a heat treatment at 220 °C for 1 h and IR spectra were collected again. During the examination, a range of wavenumber equal to 400–4000 cm^−1^, a resolution of 4 cm^−1^, and 16 scans were adopted. For each sample, the base line correction and advanced ATR correction related to the specific used crystal of diamond were carried out by Omnic Software. 

**Figure 1:**
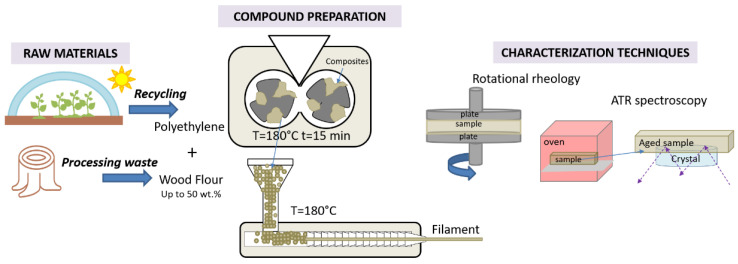
Schematic representation of compounding phase and characterization techniques.

A schematic of the experimental procedures is presented in Figure 1.

## 3. Results and Discussion

### 3.1. Torque and Temperature Measurements during Compounding Phase

**Figure 2:**
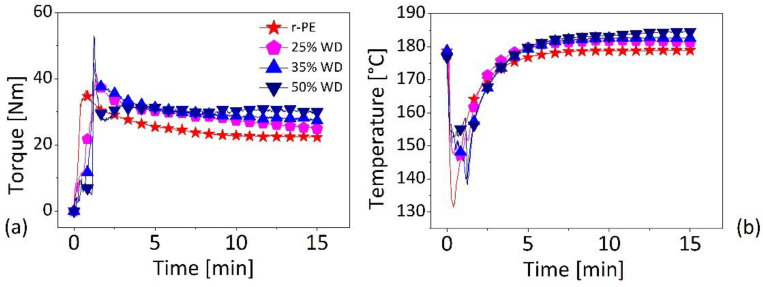
Torque and temperature measurement as a function of mixing time during the compounding phase. (Legend in Figure 2b as in Figure 2a).

During the preparation of the compounds, the torque, and the temperature were measured as a function of mixing time and the corresponding data are reported, respectively, in Figure 2a,b.

The torque (see Figure 2a) shows a non-monotonous behavior with a local maximum, due to the fact that the polymer (fed as a solid) takes a few minutes to melt, followed by decline toward a steady-state value. For all the materials the torque stabilizes after about 7–10 min and subsequently does not show any sensible decrease until the end of the compounding (i.e., 15 min), thus suggesting that mixing has taken place and no degradation of the materials is occurring.

As visible from Figure 2b the temperature of the mixing chamber, which starts from the set-point value of 180 °C, shows a sudden drop in correspondence of the loading of the materials, which are poured at room temperature. When the material are fed to the mixing chamber, they are heated and compacted, a void-free state is created, and the melting of the polymer starts at the interface with the metallic parts, which are above the melting temperature of polyethylene (140 °C–150 °C, [20]), [21,22]. The melting is an endothermic phenomenon, consisting into the breakage of crystalline regions, requiring energy. For this reason, after an initial decrease, the temperature rises to the nominal set-point value (180 °C). It should be mentioned that, while for the r-PE the initial decline in temperature is almost instantaneous, reaching approximately 130 °C, in the case of composites this temperature drop takes longer and a higher minimum (around 140 °C) is attained. This behavior is due to a lower quantity of polymer to be melted and to reduced thermal diffusivity and specific heat of the r-PE/WOOD systems which leads to a slow-down the heat transmission dynamics and to reduced energy request for the melting process [23]. 

All the systems containing the wood flour (WD) show similar behaviors of temperature evolution. It should be mentioned that the composites at the end of mixing time of 15 min achieve temperatures that are slightly higher than the set-point of 180 °C (see Table 1 for more details). This overshoot in the temperature is particularly evident for the composite containing at 50 wt.% of WD, which reaches a temperature approximately 5 °C higher with respect to the set-point. This behavior, as the higher values of the torque (also reported in Table 1), is consistent with increased viscosities of these systems (as we will see in the next paragraph) due to the friction between the wood particles in motion within the melted matrix. In other words, when the filler loading is increased, less quantity of polymer is available for incorporating particles, the inter-particles distance is reduced, and the frequency of particle-to-particle collisions increases. As the viscosity of the system increases higher torques are required for the mixing, and an increased viscous heating takes place [24]. This effect becomes more evident at high WD loadings, given an increased shear stress during the kneading of materials in the chamber and the low thermal conductivity of wood particles (0.09–0.19 W/mK) [25] compared to that of polyethylene (0.30–0.44 W/mK) [26]. In this condition, the system are less able to dissipate the energy causing mixture overheating.

According to previous a work on the compounding [27], processing variables, such as torque (M) and total mechanical energy (TME), shown in Table 1, provide useful information about the dispersion of the filler within the matrix. A comparison of the values corresponding to the matrix (r-PE alone) and to the composites show for the total mechanical energy (TME) an increase of about 14% for the composites independently of the wood content. Such a behavior suggests a poor dispersion of filler within the matrix.

### 3.2. Dynamic Rotational Rheology

#### 3.2.1. Time Sweep Tests

**Figure 3:**
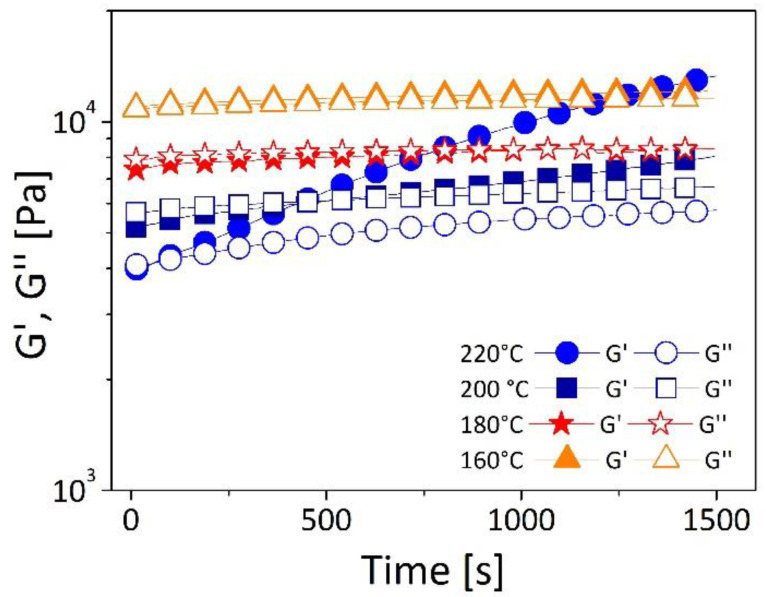
Storage (G’) and Loss (G’’) modulus as function of time and temperature for recycled polyethylene. (r-PE) Measurements were conducted at an oscillatory frequency of 1 rad/s and a strain amplitude of 1%.

Thermal stability of the matrix (recycled polyethylene, r-PE) was studied through time sweep test, performed at four different temperatures 160 °C, 180 °C, 200 °C, 220 °C. Experimental results are shown in Figure 3 in terms of storage (G’) and loss (G’’) moduli as a function of time (1500 s).

By heating from 160 °C to 220 °C, a strong change in rheological response of the resin is observed. More in details, while at 160 °C and 180 °C both G’ and G’’ are almost time-independent over the entire experiment; at the temperatures of 200 °C and 220 °C, both G’ and G’’ show and increasing behavior with G’ having faster kinetics compared to G’’.

This behavior can be attributed to a thermal degradation of the polymer that seems to be negligible at 160 °C and 180 °C and relevant at 200 °C and 220 °C. These temperatures are consistent with data from literature for both low- and high-density polyethylene. In particular Dordinejad et al. [28], who studied the degradation of low-density polyethylene by time sweep rheometry, found that the growth rates of viscoelastic moduli increase with temperature and attributed this behavior to crosslinking reactions dominating in the thermal degradation mechanism. Mariani et al. [29], who studied the correlation between processability and properties of a high density polyethylene with a rheological approach, found increasing viscoelastic moduli with time and temperature; they attributed this behavior to an increase in molecular weight of the polymer due to the creation of long-chain branching originating from the reaction between the alkyl radical, formed during polymer processing, and vinyl groups of unsaturated ends of HDPE.

**Figure 4:**
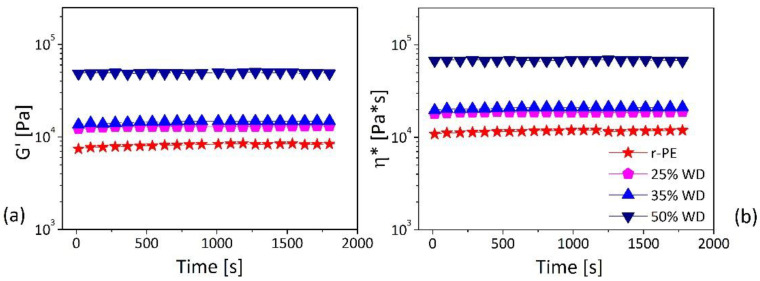
Time sweep tests performed at 180 °C (at an oscillatory frequency ω = 1 rad/s and a strain amplitude γ = 1%) for the developed formulations: (**a**) G’ and (**b**) η*.

As our matrix has proved to be stable at temperatures as high as 180 °C, the thermal stability of the composites was studied at the temperature of 180 °C. The viscoelastic properties, in terms of storage modulus (G’) and complex viscosity (η*), are reported as a function of time in Figure 4.

All the materials, also at highest filler loading (50 wt.%), show rheological properties almost time-independent for the entire duration of the experiments, thus confirming the thermal stability of the materials at temperatures as high as 180 °C. A similar behavior was observed by Mazzanti et al. [30] in the case of wood-polypropylene composites, who related the stable moduli to absence of wood degradation at a concentration of 70 wt.%. It should be mentioned that the good stability of our composites is in line with observations of Sliwa et al. [31], who found a spectacular improvement of thermal stability of wood-polymer composites due to the formation of a protective barrier of char which hinders thermo-oxidation process. Furthermore, this behavior is consistent with the formation of tortuous pathways of dispersed fillers in the polymer that slows the mass diffusion of degradation products from the polymer’s bulk to the gas phase, reduces overall the thermal conductivity, and acts as a protection against thermal decomposition observed for composites [32]. 

#### 3.2.2. Frequency Sweep Tests

**Figure 5:**
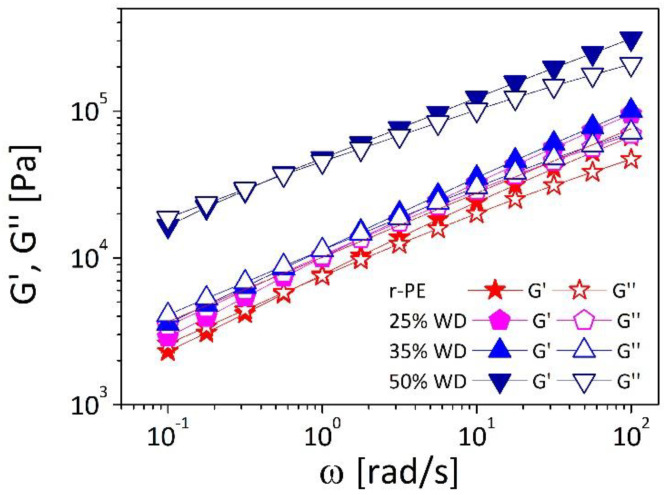
Storage (G’) and Loss (G’’) modulus vs oscillatory frequency at temperature of 180 °C for composites.

In Figure 5, the storage and the loss moduli at 180 °C are plotted, for the different wood contents, as a function of the oscillatory frequency (ω). All the materials show the typical viscoelastic behavior of entangled polymeric liquid, with the loss modulus (G’’) dominating at low frequencies, the storage modulus (G’) dominating at higher frequency, and a crossover (where G’ = G’) at intermediate frequencies. 

From the observation of Figure 5, we note that as the amount of wood is increased the viscoelastic moduli move towards higher values and the crossover shifts towards lower frequencies with respect to the r-PE thus evidencing the reinforcing effect of the filler.

More in details, it should be noticed that even for the formulation containing the highest amount of wood loadings (50% WD) a low-frequency region with a frequency-independent elastic modulus G’, typical of interconnected networks, cannot be observed. This behavior indicates poor compatibility and weak interfacial adhesion between the two phases that prevent the interactions responsible for a rheological percolation and the formation of a three-dimensional network [33,34]. On the contrary, the fact that the crossover frequency decreases as a function of the filler loadings, indicates that, despite the weak affinity between the two phases, a greater amount of particles limits the motion and slows down relaxation dynamics of polymeric chains. These results are in line with other works of wood-plastic composites [35], where an increase in G’ and G’’, an attitude of material to elastic behavior, and a crossover point moving towards lower frequency were observed with increasing filler concentration. Literature data suggest for our composites a possible improvement of melt strength [36] which could be very useful in view of the utilization of these material for the production of printed objects.

**Figure 6:**
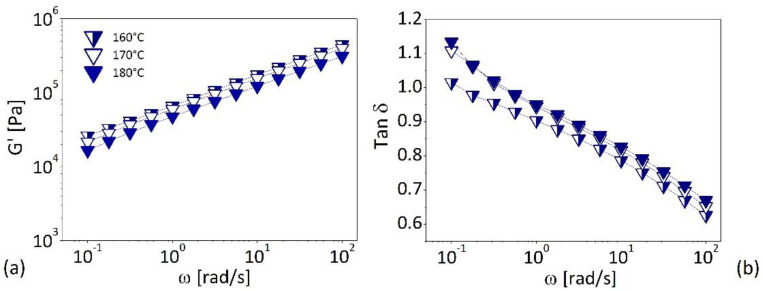
Effect of temperature on storage modulus (**a**) and dissipation factor (**b**) of composites at 50% in wt. of wood flour.

The temperature dependency of viscoelastic behavior of the wood–plastic composites is shown in Figure 6, where the storage modulus (G’) and the tangent of the phase shift angle (tan δ) are plotted as a function of the oscillatory frequency for system filled with 50 wt.% of wood (Figure 6a,b).

**Figure 7:**
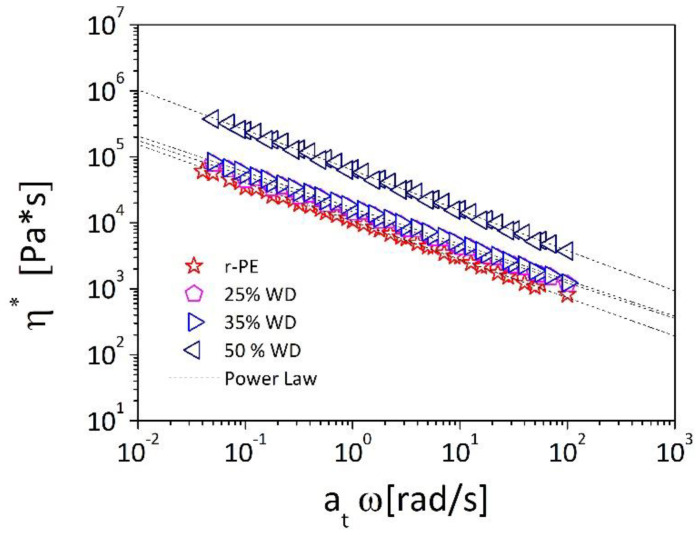
Complex viscosity against reduce frequency master-curves of composites. The dotted lines represent fittings with a power-law model (Equation (1)).

The data reported in Figure 6 and, in particular, the similarity of the G’ against ω curves (on double logarithmic scale) collected at the different temperatures, suggest that for the elastic modulus, and more generally for any viscoelastic function, the effect of the temperature can be separated by that of the frequency, and can be described by a temperature-shift factor (*a_T_*). The temperature dependence of *a_T_* represent the effect of temperature on viscoelastic properties; the values of *a_T_* are determined empirically by making tan δ curves (i.e., a density independent viscoelastic function) superimpose on the one chosen as reference. In our case, we have chosen a reference temperature (at which the shift factor is equal to unity) to be T_0_ = 180 °C. If the complex viscosity (η*) data are plotted against the reduce frequency ω·*a_T_* the master curves reported in Figure 7 are obtained. The master curves shown in Figure 6 represent the frequency dependence that would have been obtained measuring the complex viscosity over a wider frequency range at the (reference) temperature of 180 °C.

A knowledge, and a modeling, of the viscoelastic behavior of materials as a function of deformation rates and of the temperature is essential in order to adjust processing parameters and obtain a final product with good properties [37]; we derive in the following section analytical description of both variations.

The complex viscosities of our materials (see Figure 7) show a decreasing behavior upon the oscillatory frequency, a so-called shear-thinning or pseudoplastic behavior, that can be attributed to molecular alignments and disentanglements of the polymer chains at faster deformation rated. The most simple model that can be used to capture such a behavior is the power-law, or Ostwald-de Waele, model:

(1) $$\eta^{*}\left(\omega \right)=k\omega^{n-1}$$

where *k* is the flow consistency (Pa*s^n^) and *n* is the flow index.

**Figure 8:**
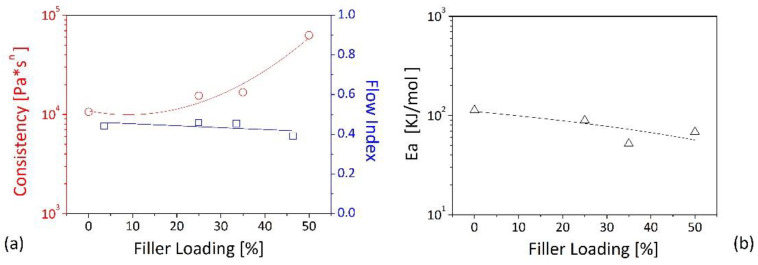
Consistency and index flow (**a**), and activation energy (**b**) against filler loading. The lines in figure are to guide the eye.

By fitting the experimental data reported in Figure 7 with Equation (1), consistency and flow index are obtained as a function of the wood concentration (Figure 8a). It can be observed that the consistency increases as a strong function of the filler loading with a 6-fold increase for the system containing 50% of wood with respect to the base r-PE. On the contrary, the flow index shows a weak dependency upon the wood contents with values around 0.4 thus suggesting the shear thinning behavior depends on the polymeric matrix and not on the filler. These results are in good agreement with the studies of Mazzanti et al. [30] and of Laufer et al. [38] who studied, respectively, of wood-PP and wood-LDPE and observed the similar variations of consistency and flow index as a function of the amount of the particles in the matrix.

The temperature dependence of viscoelastic properties is described by the shift factor *a_T_*, which at temperatures well above the glass transition temperature, can be represented by a simple Arrhenius-type equation:

(2) $$a_{T}=exp\left[\frac{E_{a}}{R}\left(\frac{1}{T}-\frac{1}{T_{0}}\right)\right]$$

where *T*_0_ is the reference temperature (in our case set to 180 °C), *E_A_* is the activation energy for the process, and *R* is the universal gas constant.

The activation energy is plotted in Figure 8b as a function the filler content for the developed compounds. It should be noted that for the matrix we have determined a value of the order of 100 kJ/mol while for the higher wood loadings we have observed values of the order of 50 kJ/mol for. As lower values of the activation energy indicate a lower sensitivity of viscoelastic properties to temperature changes, it can be affirmed that the wood-filled systems behave more in a solid-like manner also from this point of view. This behavior is consistent with literature data, even though lower values are reported for homo-polyethylene (~26 kJ/mol) and for short-chain branched PE (~34 kJ/mol). However, as it is known that long-chain branching provokes an increase in the activation energy of polyethylene, the data suggest the presence of branching in our r-PE. As stated by Park [39], the free volume is not the main aspect that dominates the macromolecular motion. At testing temperatures higher than the glass transition temperature (T_g_), the free volume is larger than that available at T_g_. In this condition, the primary fact governing the chain mobility is the thermal activation. The branching increased the activation energy of polyethylene due to the hindrance of segmental motion for interaction of long-chain branches. In other words, the higher the activation energy observed for the r-PE suggests a possible presence long-chain branches formed during processing.

The variation of complex viscosity with deformation rates, described by power-law equation (Equation (1)), allows for the calculation of viscosity at processing deformation rates, which are higher than those experimentally explored. Equation (1) can be used to adjust the processing condition for each formulation. For example, typical shear rates at gate and in the mold are in a range between 100 and 1000 s^−1^, and under such conditions, a shear-viscosity value below 1000 Pa∙s is suggested for suitable compositions [40]. Use of Equation (1) suggests that while the r-PE respects such a condition at the temperature of 180 °C while the compounds at 50 wt.% of WD requires different condition in terms of temperature (>180 °C) or shear rate (>10^3^ s^−1^).

### 3.3. Infrared Spectroscopy

**Figure 9:**
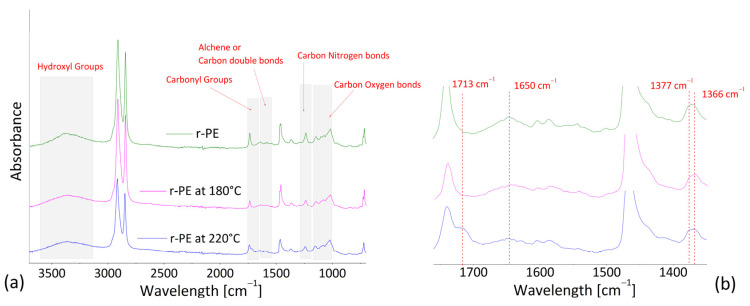
ATR spectra of recycled polyethylene (r-PE), and heated samples at 180 and 220 °C for 1800 s. (**a**) Frequency range of 800–3700 cm^−1^; and (**b**) zoom in the range 1350–1760 cm^−1^.

Infrared spectroscopy is widely used as a qualitative technique to monitor the thermal degradation of polyethylene samples. A comparison among ATR spectra of the matrix (r-PE) at room temperatures, and after heating at 180 °C and 220 °C for 1800 s, respectively, are reported in Figure 9.

For all the samples, the characteristic peaks around 2900 cm^−1^ (C–H stretching), 1460 cm^−1^ (CH_2_ deformation bending), and 720 cm^−1^ (CH rocking bending), commonly attributed to polyethylene homopolymer [41], are clearly visible. Different changes in IR spectra attributed to sample aging can be identified according to previous literature: (i) broad peaks from 3100 to 3700 cm^−1^ ascribed to hydroxyl groups; (ii) alkenes, or carbon double bonds in the range of 1600 to 1680 cm^−1^; (iii) region of 1690–1810 cm^−1^ associated to carbonyls; (iv) region at 1000–1200 cm^−1^ representative of carbon-oxygen bonds; and (v) carbon–nitrogen bond in the range of 1200 to 1280 cm^−1^. According to work of Brandon [42], usually pure polyethylene should not have nitrogen in the chemical structure, however nitrogen is present in the air and can be contained in plastic additives. 

Generally, in presence of oxygen and high temperatures, the oxidation process of polyethylene starts from weak bonds close to terminal vinyl groups and generates oxygen-containing groups, such as alcohol, ketone, acid, and so on [43]. In the samples treated at 220 °C compared to pristine r-PE, it can be noted: (i) the formation of a shoulder in correspondence of 1713 cm^−1^ (close to the peak at 1720 cm^−1^, typical of carbonyl groups), which can be correlated to the presence of ketones; (ii) the reduction in the peak at 1650 cm^−1^, which can be linked to a decrease in double bonds [41]; (iii) a small increment in intensity at 1377 cm^−1^, which can be due to the occurrence of branching. Indeed, as attested by Gulmine et al. [44], a characteristic peak at 1377 cm^−1^, typical of CH_3_ symmetric deformation, is present in LDPE and LLDPE spectra, together with peak at 1366 cm^−1^. This latter, attributed to the CH_2_ wagging deformation, is present alone in HDPE spectra. The typical peak around 909 cm^−1^ for representing changes in terminal vinyl groups was too weak to be detected accurately, as verified by Brandon et al. [42]. 

Infrared spectrum of wood flour, reported in literature [45,46], showed the predominance of hydroxyl band at 3100 to 3700 cm^−1^ and ether species (C–O–C) at 1000–1150 cm^−1^, then weak bands at 1800–1680 cm^−1^ of carbonyl groups, at 1650–1500 cm^−1^ of vinyl (C=C) groups, at 1450 and 1350 cm^−1^ due to methyl species (CH_3_).

**Figure 10:**
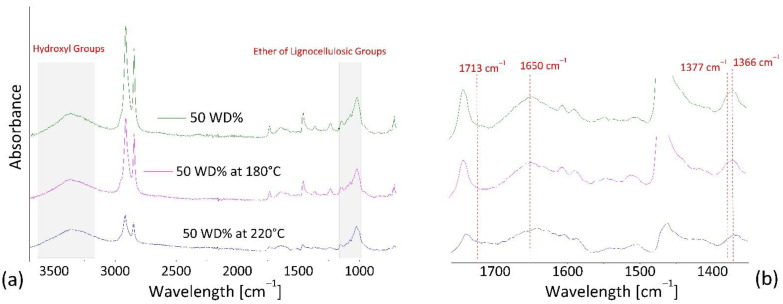
ATR spectra of composites at 50% in wt. of WD flour, and heated samples at 180 °C and 220 °C for 1800 s. (**a**) Frequency range of 800 to 3700 cm^−1^ and (**b**) zoom in the range of 1350 to 1760 cm^−1^.

By comparing spectra of the composite containing 50 wt.% of wood flour (see Figure 10) a strong decrease in intensities in the fingerprint region can be observed after the heat treatment at 220 °C. This result confirms the occurrence of thermal decomposition mechanism in wood-based compounds at temperature above 180 °C and their stability at lower temperature. Such a result is consistent with the rheological data show in the previous section.

## 4. Conclusions

In this study, we have prepared by melt blending a series of composites incorporating wood flour (25, 35, and 50% in wt.) as a filler in a recycled LDPE. The composites were characterized in terms of torque measurements performed during the mixing process, dynamic rotational rheology performed on the molten sample, and ATR spectroscopy performed on processed solid samples. Torque data have shown that the introduction of wood particles within the polymeric resin produced an increase in the overall viscosity of systems that led to sensible viscous heating phenomena during compounding. The thermal stability of the r-PE (the matrix) was verified at temperatures of 180 °C for processing times up to of 1500 s, while at higher temperatures the materials showed a rheological response characterized by time-increasing viscoelastic moduli. This behavior suggested the occurrence of crosslinking reactions governing the thermal degradation mechanism. The compounds, realized with wood loading up to 50% in wt., have shown time-independent viscoelastic properties at 180 °C, thus confirming their thermal stability at these conditions. The viscoelastic behavior of the r-PE matrix (investigated in the temperature window from 160 °C to 180 °C) suggested the presence of (short or long) branches in the macromolecular structure that were confirmed by the ATR spectra. The addition of wood particles in the r-PE matrix determined an increment in both the storage (G’) and loss (G’’) moduli, and even for the highest WD concentrations (50 wt. %) the viscoelastic spectra did not show the presence of interconnected structures. This behavior was attributed to a poor compatibility and weak interfacial adhesion between the two phases. However, the improving the elastic component in the composites due to the presence of wood flour was considered promising in view of applications to printing, and extrusion or molding technologies. Indeed, PE-wood composites seem capable of achieving good mechanical properties, processability, and durability without the use of harmful chemicals.

## Figures and Tables

**Table 1:** Processing parameters during the compounding phase: M is the average torque during the last 10 min of mixing; T is the temperature reached at the end of mixing period, and TME is the total mechanical energy (2πN∫Mdt; N = 30 rpm).

Material	M [Nm]	T [°C]	TME [kJ]
r-PE	23	179	70
25% WD	27	181	79
35% WD	28	183	81
50% WD	30	185	80

## Data Availability

The data presented in this study are available on request from the corresponding author.
